# Botulinum Toxin Injections to the Obliquus Capitis Inferioris Muscle for Dynamic Cervical Dystonia Improves Subjective Patient Outcomes

**DOI:** 10.3390/toxins16020076

**Published:** 2024-02-02

**Authors:** Robin Anne Bessemer, Mandar Jog

**Affiliations:** Department of Clinical Neurological Sciences, London Health Sciences Centre, 339 Windermere Road, London, ON N6A 5A5, Canada; mandar.jog@lhsc.on.ca

**Keywords:** dystonia, tremor, botulinum toxin, torticollis

## Abstract

The obliquus capitis inferioris (OCI) muscle is a significant driver of cervical dystonia with torticaput movements and a no–no head tremor. Limited data are available on the efficacy of OCI injections on patient outcomes. Our study aims to determine whether the botulinum toxin injection into OCI improves subjective patient quality of life in those with dystonic head tremors. A retrospective chart review was performed for 25 patients receiving injections into the OCI for a dystonic head tremor at the London Movement Disorders Clinic between January 2020 and January 2022. Toronto Western Spasmodic Torticollis Scale-2 (TWSTRS-2) subscale scores for disability and pain, TWSTRS-PSYCH scores, and the global impression of severity were extracted. The average TWSTRS-2 disability subscale change was −2.8 points (*p* < 0.003). The average TWSTRS-2 pain subscale change was −4.6 points (*p* < 0.003). The average TWSTRS-PSYCH score prior to injection was 5.6. After injection, the average score was 3.7 (*p* < 0.004). The patient self-reported average global impression of severity before injection was 7.0; after this, it was 4.2 (*p* < 0.0003). The OCI injection showed significant improvement in retrospective patient self-reported outcomes; it should be considered early in the treatment plan for cervical dystonia with a no–no head tremor.

## 1. Introduction

Cervical dystonia is an adult-onset focal dystonia causing involuntary movements of the neck, head, and shoulders [1]. Cervical dystonia is associated with high rates of musculoskeletal pain, impaired activities of daily living lower quality of life [2,3,4,5]. In the last decade, a new clinical classification scheme (the COL-CAP framework) has been utilized when evaluating cervical dystonia [6]. This framework classifies cervical dystonia into several different subtypes based on the location of action of the dystonic muscles. -Caput subtypes describe the head motion resulting from the action of muscles on the atlantoaxial joint, while -Collis subtypes describe the neck motion induced by muscle action on the cervical spine [6]. This distinction allows for a nuanced selection of muscles for treatment with botulinum toxin injections and has been associated with improved patient outcomes [7].

The torticaput subtype causes the rotation of the head on the atlantoaxial joint. It has been identified as the most common phenotype of cervical dystonia. Torticaput is often associated with additional subtypes, including laterocollis, torticollis, and laterocaput [6,8]. Dynamic dystonia, also referred to as a dystonic tremor, is commonly present in cervical dystonia patients [7,9,10]. Jost et al. (2020) demonstrated a 55.6% prevalence of dystonic head tremor in a cervical dystonia cohort [9]. In patients presenting with a torticaput phenotype, the prevalence of a dystonic head tremor is higher (64.7%) [8]. Dynamic dystonia is critical to identify, as it has been associated with less favorable responses to botulinum toxin injections. Misra et al. (2012) showed that the absence of a baseline head tremor was strongly associated with an improved response to botulinum toxin injections (33.2% responder rate vs. 23.7%, OR 1.5) [10].

Dynamic dystonia in a torticaput patient causes a fine, lateral head movement, known as a ‘no–no’ head tremor. Obliquus capitis inferioris (OCI) has been identified as one of the most common primary active muscles in this rotatory head tremor [11,12]. Schramm et al. (2017) showed that OCI was active in all patients from a cohort of 35 studied with a horizontal no–no head tremor. This has been corroborated using advanced imaging studies. Su et al. (2022) utilized SPECT imaging to show that in torticaput patients with a no–no head tremor, OCI is the most frequently involved muscle [12]. 

To date, limited data are available on the specific impact of the OCI injection on patient outcomes. One small cohort study of five patients demonstrated an improvement in patient self-rated outcomes after OCI injections [13]. Our retrospective study aimed to determine whether the botulinum toxin injection of the obliquus capitis inferioris (OCI) muscle in patients with a dystonic head tremor improves patient quality of life. Our primary outcomes include the change in the Toronto Western Spasmodic Torticollis Scale-2 (TWSTRS-2) pain and disability subscale scores, TWSTRS-PSYCH scores, and the patient-reported global impression of disease severity before and after their first OCI injection.

## 2. Results

A total of 25 charts were reviewed, including 22 female patients and 3 male patients (Table 1). All patients had a mixed dystonia, with the predominant type of motion being torticaput. The muscle injection pattern varied between patients but included a combination of obliquus capitis inferioris, splenius capitis, sternocleidomastoid, levator scapulae, trapezius, semispinalis capitis, longissimus capitis, scalenus medius, masseter, and medial pterygoid (full injection patterns are available in Supplemental Table S1). Two patients were injected with abobotulinum toxin A (Dysport), 10 with onabotulinum toxin A (Botox), and 13 with incobotulinum toxin A (Xeomin). Two patients received unilateral OCI injections; the remaining 23 patients had OCI injected bilaterally. The average initial dosing for onabotulinum toxin A was 39 U divided bilaterally; initial dosing ranged from 20 to 50 U divided bilaterally. The average initial dosing for incobotulinum toxin A was 34 U divided bilaterally; initial dosing ranged from 10 to 50 U divided bilaterally. The average initial dosing for abobotulinum toxin A was 125 U divided bilaterally; initial dosing ranged from 100 to 150 U divided bilaterally. Patients who had already been receiving injections from our clinic underwent an average of 14.5 injection cycles prior to OCI’s addition to the injection pattern. For patients who had already been receiving injections, OCI was the only new muscle added to their pre-existing injection scheme. Four patients were new consultations who had OCI included in their initial injection scheme. Of the new patients, three received incobotulinum toxin A (Xeomin), and one received onabotulinum toxin A (Botox). Electromyogram (EMG) guidance was used for all OCI injections. Ultrasound guidance was not used.

**Figure 1:**
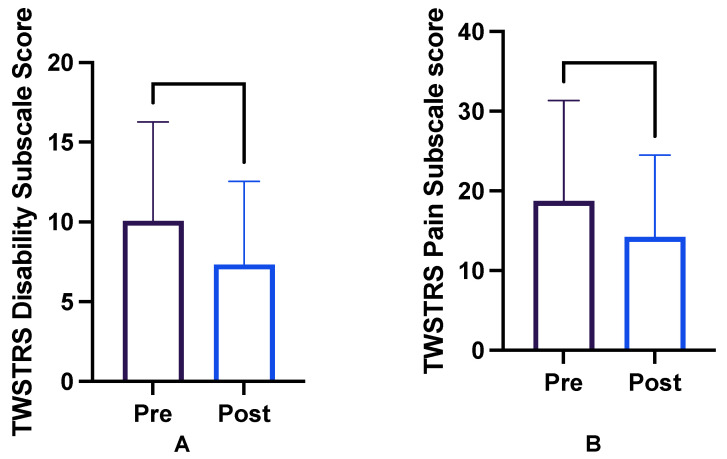
TWSTRS-2 subscale scores for (**A**) disability and (**B**) pain before and after the injection of botulinum toxin into obliquus capitis inferioris.

Scores are reported as before and after the initial injection visit in which OCI was included. The average TWSTRS-2 disability score before OCI injection was 10.1 (descriptive statistics in Table 2). After OCI injection, the average disability score was 7.3 (maximum possible score 30) [13]. The TWSTRS-2 disability subscale average change was −2.8 points (*p* < 0.003; −4.463 to −1.057; Figure 1). 

The average TWSTRS-2 pain score before OCI injection was 18.8; after OCI injection, it was 14.2 (maximum possible score 40; see Table 3 for descriptive statistics) [13]. The average pain subscale change after OCI injection was −4.6 points (*p* < 0.003; −7.447 to −1.673; Figure 1). The average aggregate change in both subscales was −7.3 points (*p* < 0.0012).

**Figure 2:**
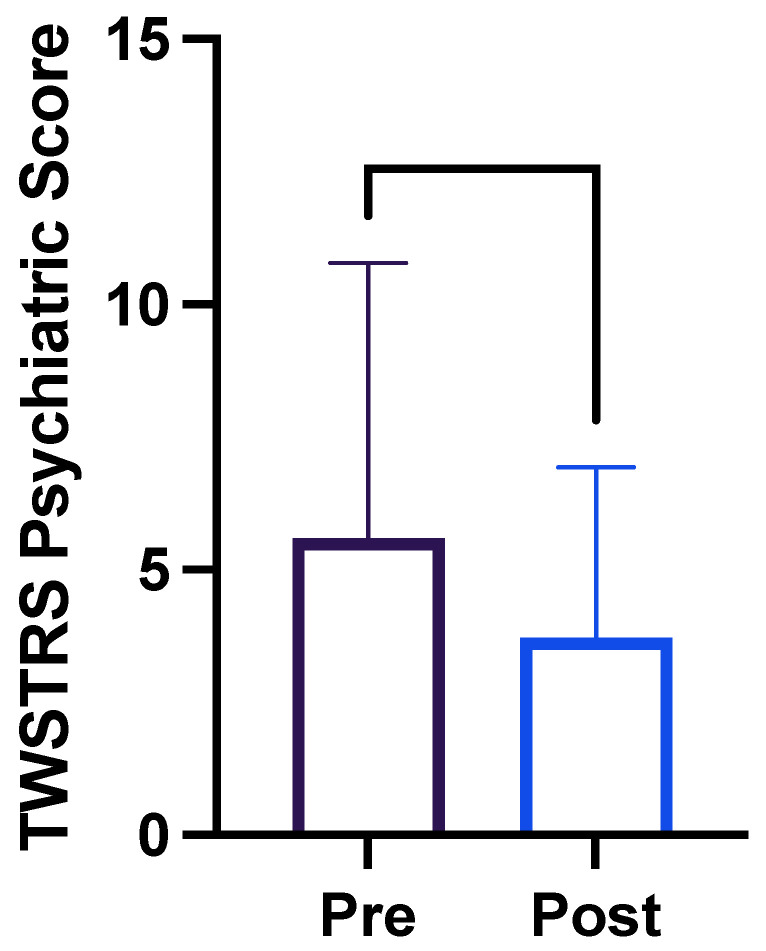
TWSTRS-PSYCH scores before and after the injection of botulinum toxin into obliquus capitis inferioris.

The average TWSTRS-PSYCH score prior to OCI injection was 5.6 (maximum possible score 24; see Table 4 for descriptive statistics). After OCI injection, the average TWSTRS-PSYCH score was 3.7. The mean change to the TWSTRS-PSYCH score was −1.880 (*p* < 0.0001, −3.440 to −0.3203; Figure 2) [13]. 

**Figure 3:**
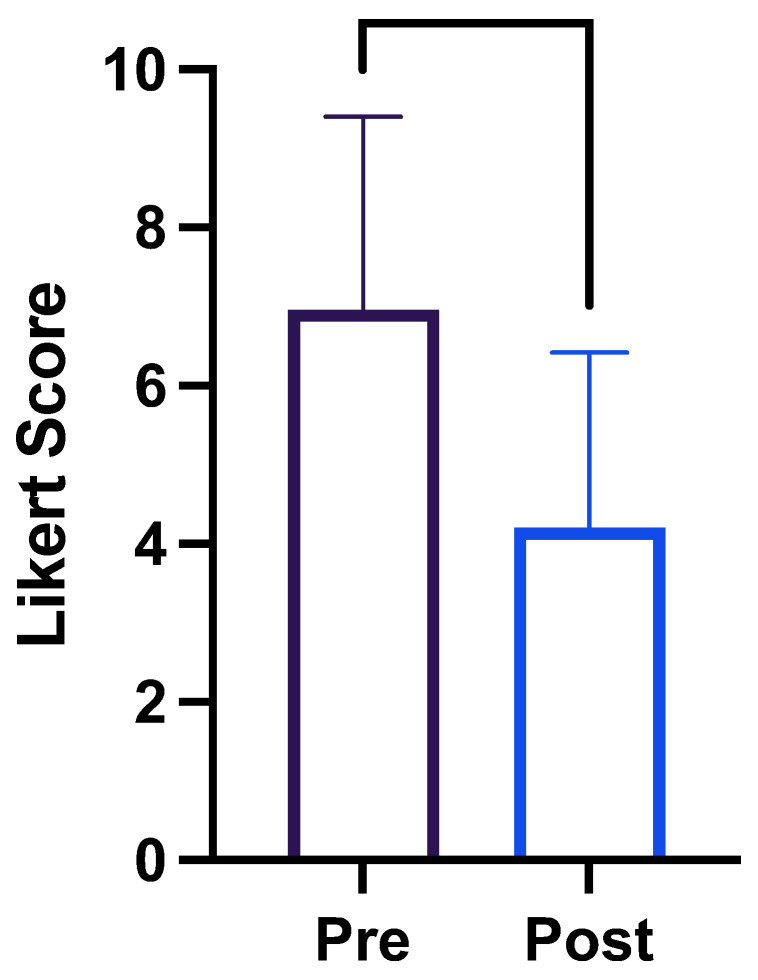
Patient global impression of disease severity before and after the injection of botulinum toxin into obliquus capitis inferioris.

Patients’ self-reported average global impression of severity (as measured on a 10-point Likert scale) before OCI injection was 7.0; after the injection, it was 4.2 (*p* < 0.0003; −3.906 to −1.047; Figure 3; descriptive statistics in Table 5).

## 3. Discussion

Recent evolution in the classification of cervical dystonia has drawn attention to the ‘caput’ movements of the head on the atlantoaxial axis. The obliquus capitis inferioris (OCI) is a key muscle driving horizontal rotational caput movements, particularly in those with a horizontal no–no head tremor [11]. OCI is a deep suboccipital muscle that originates from the lateral surface of the C2 spinous process and inserts itself on the posterior aspect of the occipital bone [14]. It lies deep in the trapezius and semispinalis capitis muscles. When activated, it produces an ipsilateral atlantoaxial rotation. 

Due to the small size and deep location of the OCI, advanced injection techniques, including ultrasound guidance, or EMG guidance are recommended. To begin injection with surface landmarks, the ipsilateral inferior margin of the pinna is identified, and a point 1 cm (approximately one fingerbreadth) below the occipital protuberance in the midline. The needle is inserted halfway between those two landmarks and travels between the lateral border of the semispinalis capitis and the upper border of the splenius capitis. To perform an EMG-guided injection, an advanced needle with a 45-degree angle in the medial direction to a depth of approximately 2 cm is used. To confirm needle placement, OCI can be activated using ipsilateral head rotation (Video S1). In our center’s experience, OCI is relatively easy to localize using EMG guidance. No serious side effects have been reported by our patient cohort. While not used in our center, an ultrasound-guided technique can also be used to directly visualize the OCI. The OCI can be visualized at the C1 level, deep in the splenius capitis and semispinalis capitis. An out-of-plane injection approach is typically used [7,15,16]. Our center most commonly injects bilateral OCI using a starting dose of 10–15 U onabotulinum toxin A per side. 

This study examined patient self-reported outcomes in the TWSTRS-2 subdomains of pain and disability, the TWSTRS-PSYCH scale, and a global impression of severity scale. Historically, a minimal change greater than or equal to 10 points on the total TWSTRS score was used to define a clinical response to botulinum toxin injections in cervical dystonia. The secondary analysis of the CD-PROBE study by Dashtipour et al. (2019) shows how a reduction of at least eight points on the TWSTRS-total score was necessary for a minimal patient global impression of change; while a reduction of at least seven points on the TWSTRS-total score was necessary for a minimal clinical impression of change [17]. In that analysis, a reduction by 11 or 10 points on the TWSTRS total score correlated to a very significant impression of change in patients and clinicians, respectively. Prior work by Espay et al. (2018) suggests that the minimal meaningful change in the TWSTRS-total score is variable depending on the baseline TWSTRS severity. Patients in the lowest quartile of pre-morbid TWSTRS-2 scores had a minimal meaningful change of 3.18 while those the highest quartile had a minimal meaningful change of 18.0 [18].

We showed statistically significant improvements in all the domains measured after the initial OCI injection was performed. Of note, due to the retrospective nature of this study, a torticollis motor severity scale was unable to be collected. This means a complete TWSTRS-2 score is not available for comparison with the literature on minimal meaningful change. It is not clear what the threshold for minimal meaningful change in each individual subdomain is. However, the statistically significant change in the patient self-reported global impression of severity in our study suggests that the OCI injections led to clinically meaningful change. 

Several patients were noted to have symptom improvement that was not captured by the TWSTRS-2 subscales. Specifically, patients noted that the horizontal rotational head tremor was particularly bothersome at night when attempting to sleep due to the fine motion of their head on the pillow. Multiple patients noted sleep improvements after OCI injection. 

This study demonstrates a benefit to patient self-reported outcomes after OCI injection for dynamic rotational dystonia. This study is limited by the small sample size, the lack of a control group, and the retrospective nature of this study. Additionally, our center uses electromyography (EMG)-guided injections for the localization of OCI, but no ultrasound. Some centers prefer to directly visualize the OCI using the ultrasound-guided approach. Without direct visual confirmation, it is possible additional muscles received the toxin. Additionally, regardless of the injection technique, the local diffusion of the toxin to neighboring muscles is possible. In the future, a prospective study with an objective quantification of tremor severity in addition to patient self-reported outcomes is warranted. Additionally, analysis over a more prolonged length of follow-up is also helpful in determining the clinical course of patients receiving this type of injection. 

The obliquus capitis inferioris should be considered for botulinum toxin injections in cervical dystonia patients with a head tremor. We recommend that the injections be performed using either EMG or ultrasound guidance. The OCI should be incorporated into injection patterns early in the treatment of cervical dystonia, given the potential for significant clinical benefit. 

## 4. Materials and Methods

A retrospective chart review was performed for 25 patients with cervical dystonia and a horizontal head tremor who were injected in the London Movement Disorders Clinic between January 2020 and January 2022. All patients received botulinum toxin injections into the OCI. We included patients who had previously been injected in our clinic for cervical dystonia but who had not achieved satisfactory symptom control with their regular injection scheme and so had OCI added to their injection pattern. We also included patients for whom OCI was selected as part of their first injection pattern due to a predominant dynamic torticaput phenotype. Patients receiving onabotulinum toxin A, abobotulinum toxin A, and incobotulinum toxin A were all included. 

Scores were extracted from the chart from the visit prior (“before”) and the next visit immediately after (“after”) the first visit where OCI was injected. Toronto Western Spasmodic Torticollis Scale-2 (TWSTRS-2) subscale scores [15] for disability and pain were extracted. Additionally, TWSTRS-PSYCH scores [13] and the global impression of severity as rated by the patient on a 10-point Likert scale were also extracted. Patient demographic information was collected, including age, the duration of injections prior to OCI, toxin type and dose, and muscle injection patterns. 

Patients were excluded if they had no TWSTRS-2 scores recorded or were lost to follow-up after injections were performed. Informed consent for chart review was obtained from all participants. This study was approved by the local ethics committee at Western University (identifier: R22-495; 10/17/2022). 

Graphpad8 software was used to perform statistical analysis. The mean disability subscale, pain subscale, TWSTRS-PSYCH scores, and global impression of severity before and after the initiation of botulinum toxin in the OCI muscle were calculated. The Shapiro–Wilk test was completed to assess normality. A paired *t*-test was performed on all parametric data sets. Non-parametric data sets were analyzed using the Wilcoxon test. A *p*-value of <0.05 was selected for statistical significance. 

## Figures and Tables

**Table 1:** Patient characteristics.

*n*	25
Female *n* (%)	22 (88%)
Male *n* (%)	3 (12%)
Average Age (years)	62
Onabotulinum toxin A *n* (%)	10 (40%)
Incobotulinum toxin A *n* (%)	13 (52%)
Abobotulinum toxin A *n* (%)	2 (8%)
OCI injected at first visit *n* (%)	4 (16%)
OCI added to injection pattern *n* (%)	21 (84%)

**Table 2:** Descriptive statistics for TWSTRS-2 disability scale.

Descriptive Statistics	Pre-Injection (*n* = 25)	Post-Injection (*n* = 25)
Mean	10.1	7.32
Minimum	0.0	0.0
Maximum	24.0	19.0
Range	24.0	19.0
Standard Deviation	6.19	5.22
Standard Error of Mean	1.24	1.04

**Table 3:** Descriptive statistics for TWSTRS-2 pain scale.

Descriptive Statistics	Pre-Injection (*n* = 25)	Post-Injection (*n* = 25)
Mean	18.8	14.2
Minimum	0.0	0.0
Maximum	40.0	33.0
Range	40.0	33.0
Standard Deviation	12.6	10.3
Standard Error of Mean	2.5	2.1

**Table 4:** Descriptive statistics for TWSTRS PSYCH scale.

Descriptive Statistics	Pre-Injection (*n* = 25)	Post-Injection (*n* = 25)
Mean	5.6	3.7
Minimum	0.0	0.0
Maximum	21.0	11.0
Range	21.0	21.0
Standard Deviation	5.2	3.7
Standard Error of Mean	1.0	0.6

**Table 5:** Descriptive statistics for the global impression of change.

Descriptive Statistics	Pre-Injection (*n* = 25)	Post-Injection (*n* = 25)
Mean	7.0	4.2
Minimum	2.0	1.0
Maximum	10.0	10.0
Range	8.0	9.0
Standard Deviation	2.4	2.2
Standard Error of Mean	0.5	0.4

## Data Availability

The data presented in this study are available in Supplementary Table S1.

## Supplementary Materials

The following supporting information can be downloaded at: https://www.mdpi.com/article/10.3390/toxins16020076/s1, Table S1: Supplementary data with injection scheme and outcomes for each patient; Video S1: Demonstration of EMG-guided injection into the right obliquus capitis inferioris muscle.
